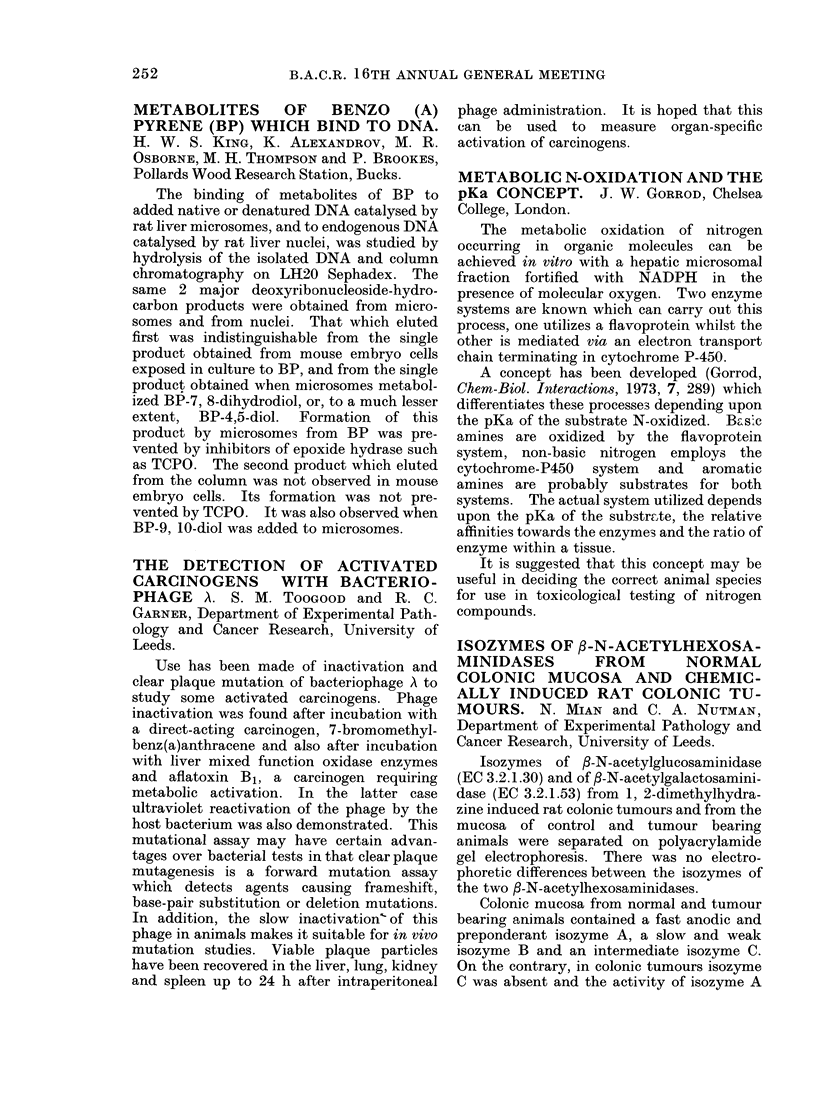# Proceedings: Metabolic N-oxidation and the pKa concept.

**DOI:** 10.1038/bjc.1975.196

**Published:** 1975-08

**Authors:** J. W. Gorrod


**METABOLIC N-OXIDATION AND THE pKa CONCEPT.** J. W. Gorrod, Chelsea College, London.

The metabolic oxidation of nitrogen occurring in organic molecules can be achieved *in vitro* with a hepatic microsomal fraction fortified with NADPH in the presence of molecular oxygen. Two enzyme systems are known which can carry out this process, one utilizes a flavoprotein whilst the other is mediated *via* an electron transport chain terminating in cytochrome P-450.

A concept has been developed (Gorrod, *Chem-Biol. Interactions*, 1973, **7**, 289) which differentiates these processes depending upon the pKa of the substrate N-oxidized. Basic amines are oxidized by the flavoprotein system, non-basic nitrogen employs the cytochrome-P450 system and aromatic amines are probably substrates for both systems. The actual system utilized depends upon the pKa of the substrate, the relative affinities towards the enzymes and the ratio of enzyme within a tissue.

It is suggested that this concept may be useful in deciding the correct animal species for use in toxicological testing of nitrogen compounds.